# 2659. Substantially Elevated Influenza Risk in Vaccinated Immunocompromised Adults and High-Risk Patients Relative to All Vaccinated

**DOI:** 10.1093/ofid/ofad500.2270

**Published:** 2023-11-27

**Authors:** Natalia Zemlianskaia, Amanda Zheutlin, Yoshihiko Murata, Jyotsna Bhattacharya, Geoffroy Coteur, Gabriela Ispas, Jorge Villacian, Jason Chien, David Hong, Jennings Xu, Breno Neri, Khaled Sarsour, Tripthi Kamath

**Affiliations:** Janssen Pharmaceuticals, Titusville, New Jersey; Janssen Pharmaceuticals, Titusville, New Jersey; Formerly Merck & Co., Inc., Rahway, New Jersey; Janssen Pharmaceuticals, Titusville, New Jersey; Janssen Pharmaceuticals, Titusville, New Jersey; Janssen Pharmaceuticals, Titusville, New Jersey; Janssen Pharmaceuticals, Titusville, New Jersey; Janssen Pharmaceuticals, Titusville, New Jersey; Janssen Pharmaceuticals, Titusville, New Jersey; Janssen Pharmaceuticals, Titusville, New Jersey; Janssen Pharmaceuticals, Titusville, New Jersey; Janssen Pharmaceuticals, Titusville, New Jersey; Janssen Pharmaceuticals, Titusville, New Jersey; Janssen Pharmaceuticals, Titusville, New Jersey

## Abstract

**Background:**

Annual influenza vaccine is universally recommended for high-risk adults, but effectiveness varies between 19-60% in the general population by season and is markedly lower for specific high-risk groups, underscoring the need for novel prophylactic interventions. Though abundant in vaccine effectiveness studies, direct evidence quantifying influenza risk in vaccinated high-risk adults is limited. We aimed to estimate incidence of post-vaccination medically attended influenza-like Illness (ILI) and subsequent hospitalization among adults with chronic diseases and immunosuppression.

**Methods:**

U.S. adults who received an influenza vaccine in influenza seasons 2015-2019 were identified using Optum commercial claims database (inpatient and outpatient). Known risk factors for influenza were assessed prior to vaccination, while medically attended ILI and subsequent hospitalization (within 30 days) were ascertained post-vaccination. Incidence rates and 95% confidence intervals (CI) were estimated for individual patient risk subgroup during the study period while maintaining sample independence across years. Relative risks were calculated using all vaccinated with or without risk as control.

**Results:**

Among 5,195,160 vaccinated adults (median age 65; 73% with ≥1 comorbid condition), annual incidence of medically attended ILI was 2.04 [95% CI 2.03-2.06] per 100 person-years and 0.25 [0.25-0.26] per 100 person-years for ILI-related hospitalization. ILI-related hospitalization risk was the highest for immunocompromised transplant patients (5.6-13.9 relative risk ratio [RR] versus all vaccinated), late-stage kidney disease (RR: 5.4-7.4), and severe chronic lung disease (RR: 4.6-5.4), with likelihood of hospitalization after ILI up to 51.3%. More than half of ILI-related hospitalizations were in individuals with chronic obstructive pulmonary disorder, heart failure, or late-stage kidney disease.

**Conclusion:**

Immunocompromised and individuals with severe chronic cardiopulmonary or renal disease retained the highest residual risk of ILI-related hospitalization despite vaccination. Additional prophylactic intervention complementing vaccination could potentially offer enhanced protection for these patients.

**Disclosures:**

**Natalia Zemlianskaia, PhD**, Janssen Pharmaceuticals: Employee|Janssen Pharmaceuticals: Stocks/Bonds **Amanda Zheutlin, PhD**, Janssen Pharmaceuticals: Employee **Yoshihiko Murata, MD, PhD**, Gilead Sciences: Current employee of Gilead Sciences|Gilead Sciences: Stocks/Bonds|Merck Research Laboratories: Listed as co inventor in a patent|Merck Research Laboratories: Former employee of Merck Research Laboratories|ViiV Healthcare: Former employee of ViiV Healthcare **Jyotsna Bhattacharya, MD, FAAP**, Janssen Pharmaceuticals: Current Employee|Janssen Pharmaceuticals: Stocks/Bonds **Geoffroy Coteur, PhD**, Janssen Pharmaceutica: Honoraria|Janssen Pharmaceutica: Stocks/Bonds **Gabriela Ispas, PhD**, Janssen Pharmaceuticals: Current Employee|Janssen Pharmaceuticals: Stocks/Bonds **Jorge Villacian, MD**, Janssen Pharmaceuticals: Current Employee|Janssen Pharmaceuticals: Stocks/Bonds **Jason Chien, MD, MS**, Janssen Biopharma: Stocks/Bonds **David Hong, MD**, Janssen: Former employee|Janssen: Stocks/Bonds|Spero Therapeutics: Employee **Jennings Xu, BA**, Janssen Pharmaceuticals: Employee|Janssen Pharmaceuticals: Stocks/Bonds **Breno Neri, PhD**, Janssen R&D: Full time employment|Janssen R&D: Stocks/Bonds **Khaled Sarsour, PhD**, Janssen Pharmaceuticals: Employee|Janssen Pharmaceuticals: Stocks/Bonds **Tripthi Kamath, PhD**, Janssen Pharmaceuticals: Employee|Janssen Pharmaceuticals: Stocks/Bonds **Xinggang lIU, MD, PhD**, Janssen Pharmaceuticals: Current employee|Janssen Pharmaceuticals: Stocks/Bonds

